# A network aggregation model for amyloid-$\beta$ dynamics and treatment of Alzheimer’s diseases at the brain scale

**DOI:** 10.1007/s00285-024-02179-5

**Published:** 2025-02-01

**Authors:** Georgia S. Brennan, Alain Goriely

**Affiliations:** Mathematical Institute, University of Oxford, Andrew Wiles Building, Woodstock Rd, Oxford, OX2 6GG UK

**Keywords:** Network model, Aggregation models, Neurodgenerative diseases, Alzheimer’s diseases, Proteinopathy, 92-10

## Abstract

Neurodegenerative diseases are associated with the assembly of specific proteins into oligomers and fibrillar aggregates. At the brain scale, these protein assemblies can diffuse through the brain and seed other regions, creating an autocatalytic protein progression. The growth and transport of these assemblies depend on various mechanisms that can be targeted therapeutically. Here, we use spatially-extended nucleation-aggregation-fragmentation models for the dynamics of prion-like neurodegenerative protein-spreading in the brain to study the effect of different drugs on whole-brain Alzheimer’s disease progression.

## Introduction

The self-assembly of proteins into ordered linear structures plays a central role in the normal functioning of organisms, spanning from bacteria to mammals (Fowler et al. 2006; Maji et al. 2009), with unique mechanical properties also eliciting potential in industrial contexts (Bleem and Daggett 2017; Knowles and Mezzenga 2016). Recent interest in protein aggregation has led to an explosion in exploratory studies across a broad spectrum of disciplines. A particularly pressing motive for such research has more medical origins; the undesired filamentous aggregation of proteins can have severe repercussions on an organism’s well-being. In such contexts, aggregation is the defining mechanism driving a cascade of pathogenic proteins characteristic of various diseases. There are now approximately 50 disorders associated with a particular class of protein filaments, known as amyloids, with disparate symptoms ranging from non-neuropathic localized amyloidosis, such as type II diabetes, to neurodegenerative diseases such as Alzheimer’s disease (AD), Parkinson’s disease and Huntington’s disease (Chiti and Dobson 2006). Despite the diversity of pathogenic proteins involved in these disorders (Chiti and Dobson 2017), experimental studies uncover commonalities in the underlying physicochemical and biochemical disease origins. This shared characteristic is the misfolding of normally soluble, functional peptides and proteins and their subsequent conversion into intractable aggregates (Knowles et al. 2014). Although only understood just decades ago neurodegenerative diseases are no longer rare, and are rapidly becoming among the most common and debilitating medical conditions in the modern world (Wortmann 2012). This growing problem poses significant challenges in modern healthcare, making any progress in our understanding of amyloid fibrillization crucial, holding implications for a wide range of debilitating medical conditions.

**Fig. 1:**
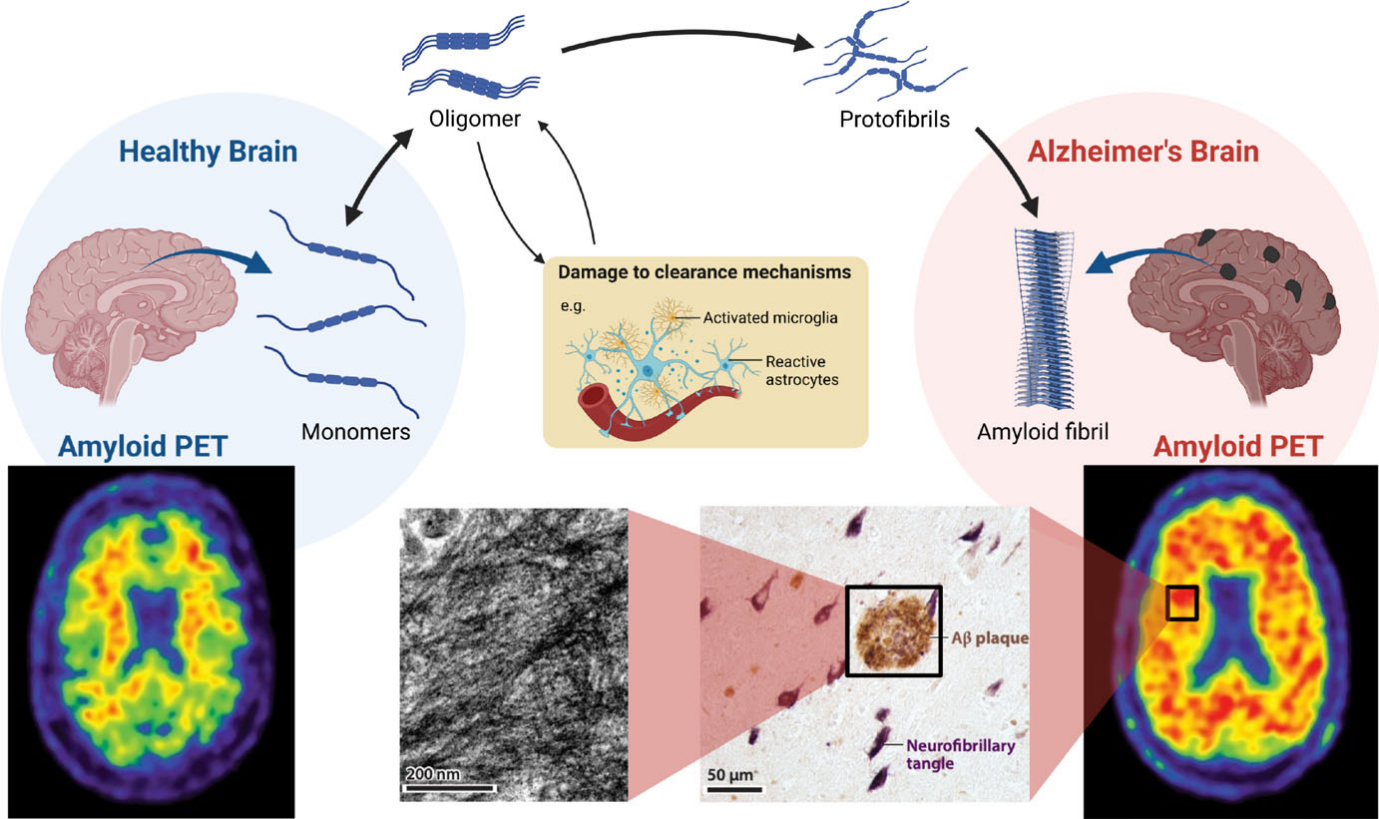
The process of aggregation, from a healthy brain to an unhealthy brain with damaged clearance and saturated in amyloid fibrils, as shown also in amyloid PET images adapted from Ten Kate et al. (2018), and also shown in a light micrograph of A$\beta$ plaques, and an electron micrograph showing a mass of extracellular amyloid fibrils in an A$\beta$ plaque at the microscopic scale, adapted from Walker and Jucker (2015)

The AD brain has defining pathological features of cortical amyloid plaques, comprising a fibrillar form of the amyloid-$\beta$ protein (A$\beta$) as depicted in Fig. 1. It is thought that such A$\beta$ accumulation facilitates the subsequent cascade of neurofibrillary tangles (NFTs) from aggregated tau protein (Lansbury 1996; Goedert et al. 2017). Several hypotheses govern the deterministic modelling of protein accumulation and propagation in AD to gain meaningful estimates of its dynamics. First, the *prion-like hypothesis* (Frost and Diamond 2010; Jucker and Walker 2018; Olsson et al. 2018; Goedert 2015; Mudher et al. 2017) broadly postulates that neurodegenerative diseases result from an accumulation of misfolded forms of these proteins, which aggregate and contribute to neurodegenerative pathology. In this process, disease-specific misfolded proteins act as a template upon which healthy proteins misfold in a manner akin to prion formation (Prusiner 1998), forming extensive chains transported through the brain along axonal pathways. Given that aggregates of differing sizes exhibit unique transport characteristics and varying toxicity levels, it is essential to monitor their spatial and temporal evolution independently. Second, the *amyloid-*$\beta$ *hypothesis* (Hardy and Higgins 1992; Hardy and Allsop 1991; Selkoe and Hardy 2016) establishes that amyloidogenic protein accumulation in AD could be causative, placing A$\beta$ central to disease pathology with supporting experimental evidence suggesting A$\beta$ as a primary driver of AD (Selkoe 2001). This hypothesis has guided most AD research over the past two decades and motivated the development of many therapeutic antibodies targeting different species of the A$\beta$ peptide (Härd and Lendel 2012). In the last decade, 15 potential therapeutics targeting the role of A$\beta$ in AD in various ways, including inhibition of enzymes involved in A$\beta$ production and removal of A$\beta$ aggregates using antibodies, have been tested in phase III clinical trials (Karran and De Strooper 2022). However, the failure of most such drug trials and recent experimental evidence has renewed scrutiny of its foundational assumptions, arguing for the possible importance of other mechanisms. Moreover, an alternative theory, the *A*$\beta$ *oligomer hypothesis*, suggests that oligomers composed of small numbers of A$\beta$ peptides are the most relevant pathological A$\beta$ species, with amyloid plaques perhaps serving as a reservoir for such species (Hong et al. 2018; Walsh and Selkoe 2020). Consistent with this, next-generation therapeutic intervention strategies targeting low molecular weight oligomers of A$\beta$ are showing promise (Linse et al. 2020).

Another potential driving factor of AD is the clearance of misfolded proteins, a somewhat elusive and powerful in vivo effect which experiments in vitro fail to replicate. The production of tau and A$\beta$ peptides is a natural process related to neuronal activity. In a healthy brain, these standard metabolic waste by-products (Rumble et al. 1989; Bacyinski et al. 2017) are removed from intracellular and extracellular compartments by several clearance mechanisms (Tarasoff-Conway et al. 2015; Xin et al. 2018). Waste proteins are broken down by enzymes, removed by cellular uptake, crossing the blood-brain barrier, or effluxing to cerebrospinal fluid compartments, eventually reaching arachnoid granulations or the lymphatic vessels. Such healthy clearance mechanisms, working in harmony, avert the buildup of toxic A$\beta$ plaques and tau NFT, but their impairment or dysfunction can lead to AD pathology. The specific descriptions of in vivo clearance mechanisms remain a topic of clinical debate; however, the kinetics enabling toxic proteins to amass into pathological aggregates can be systematically studied in vitro and coupled to dynamic clearance mathematically to simulate the naturally therapeutic, or destabilising, effect of clearance and its response to toxic aggregate mass.

Studies of the history of medicine reveal that significant progress in preventing and treating a disease typically demands a deep understanding of its underlying causes (Dobson 2013). Crucially, however, the molecular mechanisms underlying aggregate proliferation in the complex domain of the brain are still, like clearance mechanisms, poorly understood. The community acknowledges the need for a deeper understanding of molecular processes in vivo to achieve success in therapeutic strategies (Karran and De Strooper 2022). Moreover, as emphasised by Karran and De Strooper (2022), clear experiments of therapeutic hypotheses have been challenging to conduct with anti-A$\beta$ approaches: the target is not clearly defined (amyloid plaques, A$\beta$ oligomers, or monomers), the mechanism by which A$\beta$ affects cognition is unknown (direct or indirect synaptic toxicity, induction of tau pathology, neuroinflammation, or a combination of all and other effects), sometimes failures are attributed to drug administration at too late of a stage of neurodegeneration, and there is also evidence that amyloid removal is faster in patients with high baseline levels further confounding a direct comparison of antibodies (Klein et al. 2019). Mathematical modelling can contribute to a deeper understanding of these problems and suggest new approaches. Significant progress have been made in elucidating the molecular mechanisms that occur during the assembly of purified protein molecules under controlled in vitro conditions. This advance results from new experimental methods as well as better theoretical models used to analyse the resulting data. Modelling these molecular mechanisms in a disease-relevant system, including in vivo effects such as clearance and transport at the brain scale, would provide invaluable insights to guide the design of potential cures for these devastating disorders.

The goal of this study is twofold. The primary focus is to develop and analyse, both analytically and computationally, new models to study protein aggregation kinetics, including in vivo effects such as clearance in a brain region locally, then scale up to include transport on the human connectome. Second, we use our new in vivo aggregation models to test the impact of potentially therapeutic monoclonal antibodies and inform optimal treatment strategies to maximise toxic mass clearance. Our general approach is to study the size distribution of A$\beta _{42}$ aggregates, with parameters informed by experiments in a HEPES buffer (Linse et al. 2020), both locally and brain-wide using discrete aggregation equations (Fornari et al. 2019, 2020; Brennan et al. 2023), finding analytical relationships where possible and solving numerically on the brain’s connectome. The novelty of our work is exploring how the known in vitro aggregate size dynamics and the pharmodynamic effect of drugs (Linse et al. 2020; Mazer et al. 2023) scales up to affect the progression of neurodegenerative diseases in a more realistic in vivo model at the brain scale, exploring a variety of clearance profiles reflective of treatment strategies. A primary result of interest to the computational biology and pharmaceutical community will be to demonstrate that the non-trivial in vivo effects of transport and clearance of oligomers are realized with relatively simple deterministic models and couplings, leading to effects with clear physiological interpretations in neurological disease modelling. Moreover, the mathematical analysis will highlight that clearance mechanisms are crucial in destabilizing the system towards proteopathy and potentially in restabilizing the system, with implications for therapeutic intervention within the complex domain of the human AD brain.

## From microscopic models to brain-scale dynamics

Protein aggregation pathways are complex and involve multiple steps with distinct rates (Meisl et al. 2017). Advancements based on chemical kinetics have produced a deep understanding of the fundamental mechanisms underlying the formation of aggregates in ideal conditions at the microscopic scale, thereby making clearer the potential for therapeutic intervention (Frankel et al. 2019; Kundel et al. 2018). Namely, a theoretical framework of the classic nucleated polymerisation theory supplemented with secondary aggregation pathways (Cohen et al. 2011a, b, c) has been combined with systematic in vitro experiments performed under differing conditions, such as varied concentration or pH (Meisl et al. 2016; Yang et al. 2018), to reveal such mechanisms.

**Fig. 2:**
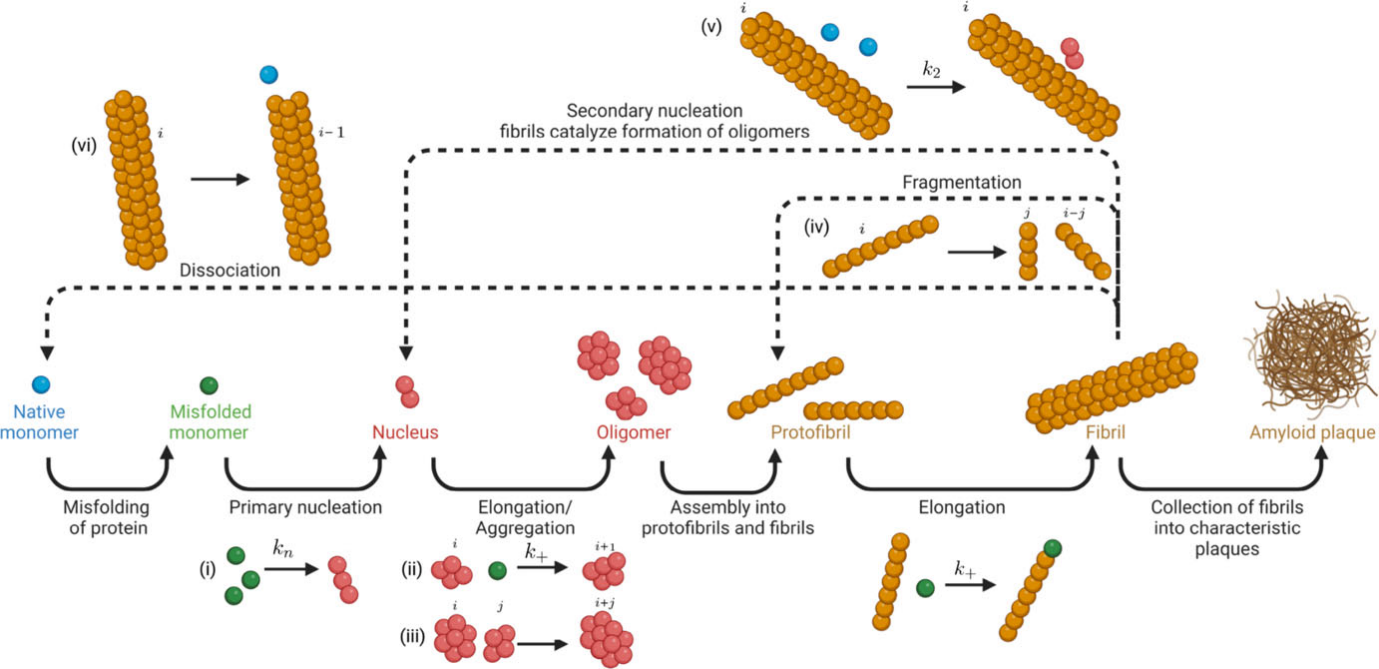
The microscopic nucleation-aggregation-fragmentation processes and secondary pathways. The respective rate constants of processes considered in our models are labelled, as detailed in Table 1

As shown in Fig. 2, it is understood that A$\beta$ monomers misfold by seeding events or prion-like templating (Nilsson et al. 2002; Jucker and Walker 2011, 2013), followed by primary nucleation, which leads to the creation of a polymer of length $n_1$ soluble monomers. Filaments elongate and dissociate linearly from both ends with the addition and removal of monomers in a reversible manner. Oligomers and fibrils of different sizes also aggregate to form larger aggregates. Additionally, secondary pathways of fragmentation and monomer-dependent secondary nucleation lead to the creation of new fibril ends from pre-existing polymers.

At present, no brain-scale in vivo therapeutic intervention model accounts for the kinetics of protein aggregation. Leading therapeutic models are in vitro models for the impact of drugs on the aggregation process (Linse et al. 2020), or brain-wide compartmental models that broadly depict disease development in the presence of drugs over months but neglect the underlying kinetics of drug action (Mazer et al. 2023). We combine the aforementioned spatial and time scales, building upon insights from the Smoluchowski-type models of Thompson et al. (2021) and Fornari et al. (2020), which respectively capture the physiological effects of clearance and brain-wide neuronal transport of aggregates.

Our general approach is to start with a microscopic model with parameters validated experimentally by in vitro experiments and extend to both in vivo settings and over the entire brain.

First, the microscopic model that we start with has been carefully calibrated in a series of experiments. In particular, it has been shown that the main processes sufficient to explain the mass increase of oligomers are linear aggregation, primary, and secondary nucleation (Frankel et al. 2019). Similarly, it has been shown that both depolymerisation and fragmentation can be ignored in the first instance (Cohen et al. 2013, 2011c). Furthermore, fibril association is considered unimportant for many protein polymers, as it is likely a slow process due to detailed structural constraints (Cohen et al. 2011a). Also, whilst there is likely variation in reaction constants with aggregate size, we consider them to be constant, constituting ensemble-averaged quantities into which small heterogeneities are subsumed.

Second, we extend the microscopic model to include in vivo mechanisms: the natural production of monomers, the clearance of monomers, and a general slowing down due to local cellular effects. This second model represents the local dynamics of oligomers in a given brain region.

Third, we extend the model to include spatial effects at the brain scale. This is performed by coupling different brain regions and assuming that proteins are mostly transported through the brain along axonal pathways. This final system belongs to the general class of network diffusion-aggregation-fragmentation models discussed in Fornari et al. (2020). In this way, we derive an entirely mechanistic brain-wide network model of neurodegeneration in which we simulate and optimize therapeutic intervention.

### A minimal microscopic model

Our minimal microscopic model for protein aggregation includes the following mechanisms: primary heterogeneous nucleation; secondary nucleation; linear elongation. Each aggregate of a given size is represented by a population. Let $p_i=p_{i}(t)$ be the concentration of aggregates of size $i = 1,2,3,\dots$. Then, the *microscopic* *master* equations are:

1a $$\begin{aligned} &  {\frac{{\text {d}}{{m}}}{{\text {d}}t}} =-2 k_{n}-2 k_{+}m P-2 k_{2} \sigma (m) m^2 M,\end{aligned}$$

1b $$\begin{aligned} &  {\frac{{\text {d}}{{p_2}}}{{\text {d}}t}} =k_{n} -2 k_{+}m p_{2}+k_{2} \sigma (m) m^2 M,\end{aligned}$$

1c $$\begin{aligned} &  {\frac{{\text {d}}{{p_i}}}{{\text {d}}t}} = 2 k_{+}m (p_{i-1}-p_{i}),\quad i>2 \end{aligned}$$

where

2 $$\begin{aligned} \sigma (m) = \frac{K_m }{K_m + m^{2}}, \quad P=\sum _{i=2}^{\infty } p_{i}, \quad M=\sum _{i=2}^{\infty } i p_{i}, \end{aligned}$$

where $m=p_1$ is the concentration of monomers, nuclei are taken to be dimers $p_2$, and the kinetic rate constants are defined in Table 1. Here, *P* and *M* are the first two moments of the population distribution; they represent the total number and total mass of aggregates, respectively.

**Table 1:** Typical parameters for the A$\beta$ model, shared by the authors of Linse et al. (2020)

Param	Mechanism	A$\beta$42 HEPES (Linse et al. 2020)	Units
$k_n$	primary nucleation	$1.6 \times 10^{-11}$	M h$^{-1}$
$k_2$	secondary nucleation	$2.1 \times 10^{14}$	M$^{-2}$h$^{-1}$
$K_m$	monomer saturation	$2.3 \times 10^{-17}$	M$^{2}$
$k_+$	elongation	$1 \times 10^{10}$	M$^{-1}$h$^{-1}$
$m_0$	initial monomer c	$3 \times 10^{-6}$	M

HEPES refers to the buffer used for the experiments

It is of interest, before we consider other effects, to understand the overall behavior of this system. This can be easily accomplished by looking at the moment equation obtained as a closed system for *m*, *M* and *P*:

3 $$\begin{aligned} &  {\frac{{\text {d}}{{P}}}{{\text {d}}t}} \,\,= k_{n}+ k_{2}\, \sigma (m) M, \end{aligned}$$

4 $$\begin{aligned} &  {\frac{{\text {d}}{{M}}}{{\text {d}}t}} =2 k_{n}+{2 k_{+}m P} + {2}k_{2}\, \sigma (m) M, \end{aligned}$$

5 $$\begin{aligned} &  {\frac{{\text {d}}{{m}}}{{\text {d}}t}} \,=-\,2 k_{n}-{2 k_{+}m P}-{2} k_{2}\, \sigma (m) M. \end{aligned}$$

**Fig. 3:**
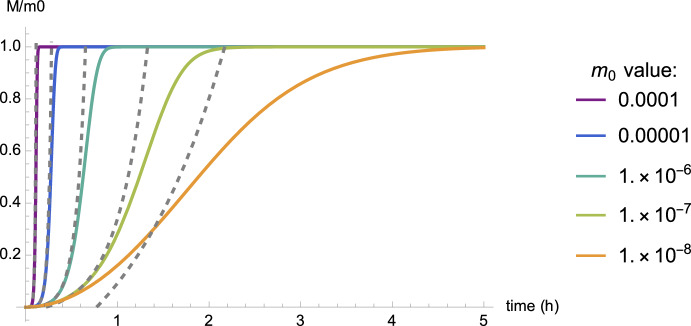
Dynamics of the microscopic minimal model based on parameters in Table 1 for various initial monomer concentrations $m_0$. For large variations of the initial monomer concentration, the dynamics is saturated within hours. The dashed curves show the linear approximation (8) from which we derive an analytical half-time $\tau _{\text {lin}}$

By construction, the total mass of the system is conserved in the in vitro model and assuming $M(0)=0$ and $m(0)=m_0$, we have $m_0=M(t) + m(t)$ for all time. As shown in Fig. 3, the behavior of this system is rather simple. The toxic mass *M* increase from 0 to $m_0$ in a sigmoid-like manner. Various approximations of this curve have been proposed and used for model validation and parameter fitting (Meisl et al. 2014; Frankel et al. 2019; Cohen et al. 2013). We observe that for large variations of the initial concentration, the dynamics saturates within hours.

To better understand the time scale involved in the process, we defined the halftime $\tau$ to be the the time at which an initial unseeded system reaches half of the final concentration $M(\tau )=m_0/2$. To approximate $\tau$, we assume that $k_n$ is small compared to other rates and linearize the system (3–4) with $m(t)=m_0-M(t)$ around $M(0)=P(0)=0$. Expanding $M=\varepsilon \tilde{M}+\mathcal {O}(\varepsilon ^2),P=\varepsilon \tilde{P}+\mathcal {O}(\varepsilon ^2)$, where $k_n=\varepsilon \tilde{k}_n$, we obtain, to first order

6 $$\begin{aligned} &  {\frac{{\text {d}}{{\tilde{P}}}}{{\text {d}}t}} \,\,= k_{n}+a \tilde{M},\quad {\frac{{\text {d}}{{\tilde{M}}}}{{\text {d}}t}} =2 k_{n}+(b-a) \tilde{P} + {2}a\tilde{M}, \end{aligned}$$

where

7 $$\begin{aligned} a=\frac{k_2 m_0^2 K_m}{K_m+m_0^2},\qquad b=2 m_0 k_{+}+\frac{k_2 m_0^2 K_m}{K_m+m_0^2}. \end{aligned}$$

A very good approximation of the exact solution of this linear system with unseeded initial conditions $\tilde{M}=\tilde{P}=0$ is obtained by neglecting the fast decaying exponential:

8 $$\begin{aligned} M(t)\approx \frac{k_n}{2a} {\left( \left( 1+\sqrt{\frac{a}{b}}\right) e^{t(a+\sqrt{ab}) t}-2\right) }. \end{aligned}$$

The linear solution gives a reasonable estimate of the initial dynamics and is simple enough to provide an analytical estimate of the halftime:

9 $$\begin{aligned} \tau _{\text {lin}}=\frac{1}{a+\sqrt{a b} } \log \left( \frac{\sqrt{b}(a m_0+2 k_n)}{k_n(\sqrt{a}+{\sqrt{b}})}\right) . \end{aligned}$$

In the range of parameters involved, we can further simplify this expression to

10 $$\begin{aligned} \tau _{\text {lin}}\approx \frac{1}{ \sqrt{2 k_+ k_2 K_m m_0}}\log \left( \frac{k_2 K_m m_0}{k_n}+2\right) . \end{aligned}$$

This solution is valid in the limit $m_0\gg K_m$, and for similar half time results given different governing equations, see Cohen et al. (2011a, 2013).

**Fig. 4:**
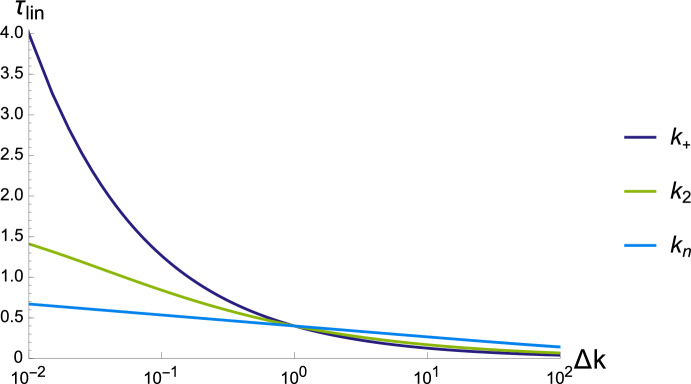
Variations of halftimes $\tau _{\text {lin}}$ due to a change of each parameter of the form $k\rightarrow (\Delta k) k_{\text {ref}}$, around the reference value $k_{\text {ref}}$ given in Table 1 (and $m_0=10^{-6}$ M). Note that in this range of parameters, the expressions (9) and (10) for $\tau _{\text {lin}}$ are indistinguishable

We now turn to the variation of time scales associated with the various parameters. We fix the initial concentration to $m_0=10^{-6}$M and systematically vary each parameter over four orders of magnitude around their values given in Table 1, to study which rate constant has the most effect on the dynamics. From (10), we see that variations in $m_0$, $k_2$ or $K_m$ are equivalent as these parameters only enter as a product. As expected, the variations due to a reduction in the primary nucleation terms are weak (and only due to the logarithm), whereas reductions in $k_2$ (with a log dependence divided by a root) are faster and variations in $k_+$ dominate (as they depend only on the square root) (Fig. 4).

The overall conclusion is that the pure conversion process of a population of monomers into oligomers is governed in vitro by rate constants that provide a typical time scale of hours, even when kinetic rates are modified by orders of magnitude. However, we know that in vivo any neurodegenerative disease evolves on time scales of years or decades. Even in the case where all kinetic rates are reduced by a factor *T*, the resulting new halftime is simply $\tau T$, which would require a factor of about $10^4$ to reach a time scale of a year. This apparent contradiction requires new mechanisms to explain the dramatic slowdown of these processes.

### A local model including clearance, production, and saturation

Based on our understanding of the minimal in vitro model that represents a closed-system, we can now extend it to include effects that appear in vivo. First, based on experimental observations in mice with prion disease (Mays et al. 2015) and in an approach adopted previously by Thompson et al. (2021), we assume that there is a regulation mechanism for the production of monomers such that their concentration remains mostly constant, despite their uptake in the formation of larger structures. Therefore, within the modelling framework, we take $m_0$ to be either constant in time or a given function depending on aging and other environmental factor with slow time variation. Second, the constant monomer assumption is supplemented with a change in the secondary nucleation term; we assume that the main autocatalytic mechanism of secondary nucleation is saturated with respect to the total mass *M* (rather than *m* in the initial model) and that the mass saturation constant is the same as the monomer saturation constant, that is, $K_M = K_m$. This assumption reflects a mechanism that restricts access to toxic mass, which is an essential consideration in the saturation of toxic mass since it has been observed in vitro that secondary nucleation is a Michaelis-Menten-type reaction (Dear et al. 2020). Third, it is believed that clearance is important in both the initiation and evolution of the disease. Therefore, we assume a linear clearance model with clearance parameters $\lambda _i$ proportional to the oligomer concentration. Assuming, in the first instance, that clearance does not evolve in time, we have

11a $$\begin{aligned} &  {\frac{{\text {d}}{{m}}}{{\text {d}}t}} =0,\end{aligned}$$

11b $$\begin{aligned} &  {\frac{{\text {d}}{{p_2}}}{{\text {d}}t}} =-\lambda _2 p_2+k_{n}m^2 -2 k_{+}m p_{2}+k_{2} \sigma (M) m^2 M, \end{aligned}$$

11c $$\begin{aligned} &  {\frac{{\text {d}}{{p_i}}}{{\text {d}}t}} = -\lambda _i p_i+ 2 k_{+}m (p_{i-1}-p_{i}),\quad i>2 \end{aligned}$$

where

12 $$\begin{aligned} \sigma (M) = \frac{K_M }{K_M + M^{2}}, \quad P=\sum _{i=2}^{\infty } p_{i}, \quad M=\sum _{i=2}^{\infty } i p_{i}. \end{aligned}$$

Additionally, as we introduce in vivo effects, heterogeneous primary nucleation on surfaces is no longer appropriate; instead, we now consider $k_n$ to represent only homogeneous primary nucleation. We note that in experiments, the process is initiated by seeding with a small amount of oligomers. If dimers are used for seeding, then the initial conditions is simply $m(0)=m_0\,P(0)=p_{2}(0), M(0)=2 p_{2}(0)$ and $k_n$ can be taken to be identically vanishing in excellent approximation for $k_n$ sufficiently small. Indeed, once the process is seeded (either through nucleation or oligomer addition), the contribution of the production term $k_n$ becomes negligible in the dynamics. Mathematically, this approximation has the advantage to have $M=P=0$ is a fixed point of the system. Therefore, in the rest of this section, we take this point of view and set $k_n=0$. Further, in the particular case where clearance is size-independent ($\lambda _i=\lambda> 0,\ i>1$), the moment equations read:

13a $$\begin{aligned} &  {\frac{{\text {d}}{{P}}}{{\text {d}}t}} \,\,= -\lambda P + k_{2}\, \sigma (M) m_0^2 M, \end{aligned}$$

13b $$\begin{aligned} &  {\frac{{\text {d}}{{M}}}{{\text {d}}t}} =-\lambda M+{2 k_{+}m_0 P} + {2}k_{2}\, \sigma (M) m_0^2 M. \end{aligned}$$

This system has two fixed points $P_1=M_1=0$ and

14 $$\begin{aligned} P_2= &  \frac{\sqrt{K_M \left( -\lambda ^2+2 \lambda k_2 m_0^2+2 k_+ k_2 m_0^3\right) }}{2 \left( k_+ m_0+\lambda \right) },\nonumber \\ \quad M_2= &  \frac{\sqrt{K_M \left( -\lambda ^2+2 \lambda k_2 m_0^2+2 k_+ k_2 m_0^3\right) }}{\lambda }, \end{aligned}$$

which exists only if

15 $$\begin{aligned} 0<\lambda <\lambda _{\text {crit}}=k_2 m_0^2+\sqrt{2 k_2 m_0^3 \left( k_2 m_0+ k_+\right) }. \end{aligned}$$

**Fig. 5:**
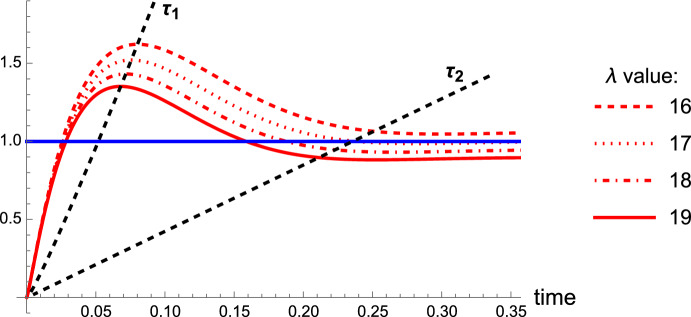
Time evolution of the oligomer toxic ratio $M/m_0$ (red) compared to initial monomer concentration $m_0/m_0$ (blue) in (12). The system is seeded with $p_2(0) = m_0\times 10^{-4}$. Parameter values are given in Table 1 with $k_n = 0$. In this case $\lambda _{\text {crit}} = 12705$ from (15). Timescales of interest $\tau _1$ and $\tau _2$ are shown in (b) by the black dashed curves (color figure online)

We conclude that this model exhibits a transcritical bifurcation with the property of having the zero trivial state stable for $\lambda >\lambda _{\text {crit}}$ and replaced by a non-vanishing oligomer concentration for $0<\lambda <\lambda _{\text {crit}}$ as shown in Fig. 5. We observe that the critical clearance is independent of the saturation constant $K_M$ and that the asymptotic mass $M_2$ scales with that constant, giving the typical size allowable local oligomer load.

**Fig. 6:**
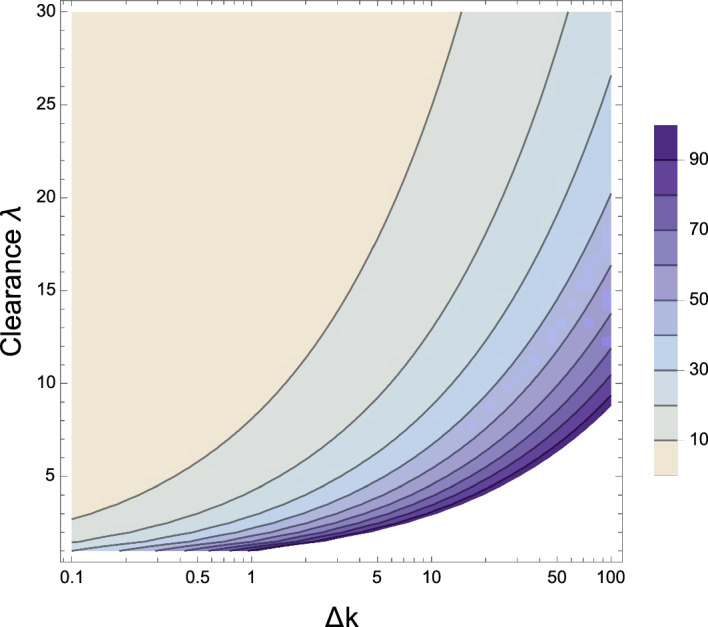
Contour plot of $M_{\text {max}}$ for varying $\lambda$ and $k_2 = k_2 \Delta k$, where $\Delta k$ varies over three orders of magnitude from $10^{-1}$ to $10^2$

With the introduction of non-zero constant clearance $\lambda$ in the system (13), there are now two timescales associated with the dynamics: a period of growth up to the maximum toxic mass at time $\tau _1\approx 1/\nu _1$ where $\nu _1 = \sqrt{2k_{+}m_0}-\lambda$ is the first eigenvalue of the system (13) when linearized, and a period of decay to time $\tau _2$ when $M-M_2=\epsilon$ where $\epsilon \ll 1$ (Fig. 5). Characteristic time scales for the amplification of the aggregate mass are obtained numerically and displayed in Fig. 6. The impact of varying $k_2$ and $\lambda$ on $M_2$ can also be seen by (14). The dependencies of both timescales on clearance and secondary nucleation are similar. Specifically, we observe that the impact of varying clearance on the timescales and toxic load in the system is profound.

Following the lag period, toxic mass *M* is dominated by elongation and secondary nucleation, up to time $\tau _1$ when the dampening effects of clearance and saturation of secondary nucleation begin to dominate, initiating a decline in toxic mass from $M_{\text {max}}$ to the steady state $M_2$. The peak toxic mass $M_{\text {max}}$, fixed point $M_2$, and associated timescales $\tau _1$ and $\tau _2$, decrease with increased clearance. Increased secondary nucleation rates $k_2$ has an effect of increasing $M_{\text {max}}$ and $M_2$ and decreasing $\tau _1$ and $\tau _2$, as expected from (14). Unless $k_2$ is altered dramatically, lowered by one order of magnitude, it has a marginal effect on lowering $\tau _1$ and $\tau _2$ with more effects in lowering $M_{\text {max}}$ and $M_2$.

Increasing clearance $\lambda$ has a more significant impact on decreasing toxic load in the local brain system (11) and both timescales $\tau _1$ and $\tau _2$, and thus dominates in the preferential dampening effects at each phase of the disease cascade. Note that this model does not capture the full disease timescales in a human brain; transport and dynamic clearance have significant effects, as incorporated in the following sections.

If a steady state $p_i^*$ for $i\ge 2$ exists (i.e. if $\lambda _i$ is below some critical clearance threshold and *M* is bounded), setting (11c) to zero, it must satisfy the recurrence relation

16 $$\begin{aligned} p_i^* = \delta _i p_{i-1}^*,\quad \delta _i = \frac{2k_+ m_0}{\lambda _i + 2k_+ m_0},\quad i>2, \end{aligned}$$

where each recursive effect is dependent on *i*. Consequently, the fixed point of the *i*-mer is

17 $$\begin{aligned} p_i^* = \triangle _i p_2^*, \quad \text {where } \quad \triangle _i = {\left\{ \begin{array}{ll} \prod _{j=3}^i \delta _j & \text {for } i>2, \\ 1 & \text {for } i = 2. \end{array}\right. } \end{aligned}$$

By definition,

18 $$\begin{aligned} M_* = \sum _{k=1}^\infty k \triangle _k p_2^* = \triangle p_2^* \end{aligned}$$

where we define $\triangle = \sum _{k=2}^\infty k \triangle _k$. Using this in solving (11b), we obtain

19 $$\begin{aligned} p_2^*= \frac{\sqrt{23} \sqrt{-\lambda _2 - 2 k_{+} m_0 + k_2 m_0^2 \triangle }}{1000 \sqrt{ \lambda _2 \triangle ^2 + 2 k_p m_0 \triangle ^2}}. \end{aligned}$$

Thus, a fixed point exists if the following three conditions are satisfied:

20 $$\begin{aligned}&\text {(C1)}\quad \lim _{{i \rightarrow \infty }} \triangle _i = 0,\end{aligned}$$

21 $$\begin{aligned}&\text {(C2)}\quad \triangle = \sum _{k=2}^\infty k \triangle _k \quad \text {converges},\end{aligned}$$

22 $$\begin{aligned}&\text {(C3)}\quad -\lambda _2 - 2 k_{+} m_0 + k_2 m_0^2 \triangle > 0 \end{aligned}$$

where (C1) reflects that the final concentration of aggregates must be decreasing with *i*, (C2) $M_\infty$ must be bounded, and (C3) requires $p_2^*\in \mathbb {R}$. Following methods presented in Thompson et al. (2021) we obtain the following analytical critical clearance formulae dependent on the form of clearance. This case is particularly interesting since, in the derivation of steady states, we see that a critical clearance can be established for different forms of size-dependent clearance. Remarkably, our results suggest that, depending on the specific size dependence, the processes of elongation and secondary nucleation contribute to the value of the critical clearance to different degrees. An important implication is that, depending on the specific mechanism of clearance, inhibition of aggregation should target different processes to reduce the critical clearance rate.

First considering constant clearance, as in (15), (C1–C3) are satisfied for

23 $$\begin{aligned} \lambda <\lambda _{\text {crit}}=k_2 m_0^2+\sqrt{2 k_2 m_0^3 \left( k_2 m_0+ k_+\right) }. \end{aligned}$$

**Fig. 7:**
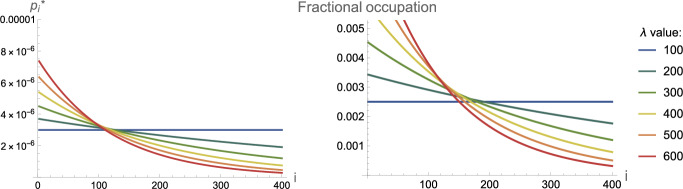
The analytical result for the equilibrium length distribution of $p_i^*$ (17) in (11) is shown for various constant clearance rates, with parameters applied from Table 1. Heightened clearance has the least impact on oligomers

The size distribution for aggregates at equilibrium $p_i^*$ for varying subcritical constant clearance rates reveals that the relative occupation of oligomers in the region appears invariant to clearance. Increasing clearance uniformly in the constant clearance regime targets larger aggregates as seen in Fig. 7. This is consistent with Oosawa theory (Oosawa and Asakura 1975; Oosawa 1970), which predicts that the length distribution initially develops into a Poisson distribution in the time taken for the monomer-polymer equilibrium to be established before relaxing over a longer time scale into an exponential distribution (Cohen et al. 2011c, [Figure 5]). Due to the constant supply of monomers, oligomer concentrations are relatively higher, reflective of a brain region.

Considering size-dependent clearance $\lambda _i$, the infinite system (11) does not yield a closed system for the moments. Still, the fixed point solution (16) enables us to determine steady states for aggregate concentrations, subject to conditions for the existence of such a steady state (C1)–(C3). These existence conditions also facilitate the identification of the bifurcation point in clearance, $\lambda _{\text {crit}}$, above which aggregation is negligible.

First, consider clearance that increases with aggregate size, $\lambda _i = i\lambda _0$. The biological understanding of this case might correspond to clearance mediated by drugs with preferential binding towards larger aggregates. In this case,

24 $$\begin{aligned} \lambda _{0,\text {crit}} = \frac{k_{+}k_2 m_0^3 - 1}{k_+ m_0 - k_2 m_0^2}. \end{aligned}$$

Since $\lambda _i$ is increasing with aggregate size like $\lambda _i = \lambda _0 i$, this intuitively suggests that a lower $\lambda _0$ is required to avoid a diseased state.

For comparison, we consider the opposite case where clearance decreases with aggregate size $\lambda _i = \lambda _0/i$ with the biological interpretation that aggregates become more difficult to clear as they increase in size. Then, an analysis of (C1)–(C3) reveal a critical clearance of

25 $$\begin{aligned} \lambda _{0,\text {crit}} = \frac{2a(a + 4k_2 m_0^2)}{a+k_2m_0^2}, \end{aligned}$$

which can be further simplified by again noting that $a = 2 k_{+} m_0 \gg k_2 m_0^2$, so we obtain the approximation $\lambda _{0,\text {crit}} \approx 2a$.

### A local model including aging and damage

As the mass of toxic proteins increases, it induces local damage that affects the proper function of the vasculature and all clearance mechanisms (Bennett et al. 2018; Carrillo-Mora et al. 2014; Canobbio et al. 2015; Michalicova et al. 2020). To capture these effects, we let the clearance rates evolve in time up to a lower minimal clearance $\mu _i,\ i>1$. The full system of equations now reads

26a $$\begin{aligned} &  {\frac{{\text {d}}{{m}}}{{\text {d}}t}} =0,\end{aligned}$$

26b $$\begin{aligned} &  {\frac{{\text {d}}{{p_2}}}{{\text {d}}t}} =-\lambda _2 p_2+k_{n}m^2 -2 k_{+}m p_{2}+k_{2} \sigma (M) m^2 M,\end{aligned}$$

26c $$\begin{aligned} &  {\frac{{\text {d}}{{p_i}}}{{\text {d}}t}} = -\lambda _i p_i+ 2 k_{+}m (p_{i-1}-p_{i}),\quad i>2,\end{aligned}$$

26d $$\begin{aligned} &  {\frac{{\text {d}}{{\lambda _i}}}{{\text {d}}t}} =\beta _i M (\mu _i-\lambda _i), \end{aligned}$$

with moment equations given by

27a $$\begin{aligned} \frac{\text {d} P}{\text {d}t}&= -\sum _{i=2}^{\infty } \lambda _i p_i + k_n m^2 + k_2\sigma (M) m^2 M,\end{aligned}$$

27b $$\begin{aligned} \frac{\text {d} M}{\text {d}t}&= -\sum _{i=2}^{\infty } \lambda _i i p_i +2k_n m^2 + 2k_{+} m P + 2k_2\sigma (M) m^2 M. \end{aligned}$$

All variables and parameters are given in Table 1, and we henceforth consider the value and form of $\mu _i$. The dynamics of this system also display a bifurcation and requires sufficient seeding before it bifurcates to a non-trivial solution where $\lambda _i\rightarrow \mu _i$ as explained in Brennan et al. (2023).

**Fig. 8:**
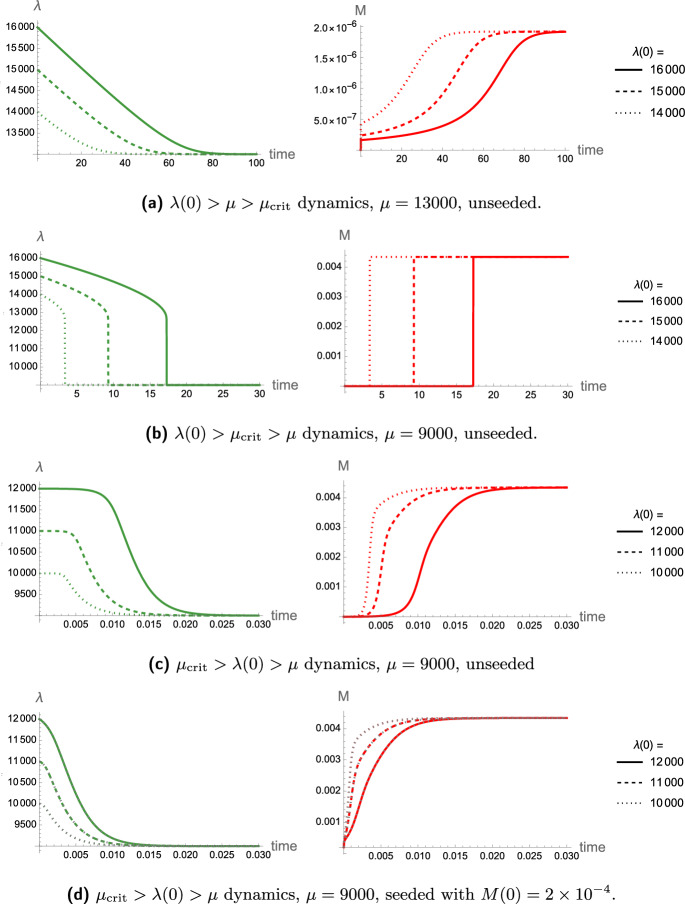
Toxic mass and clearance dynamics of (26) corresponding to different uniform initial clearance $\lambda (0)$ and basal clearance capacity $\mu$ values across aggregate sizes. Different parameter regimes are displayed relative to the critical basal clearance capacity $\mu _{\text {crit}}$, with the dynamics of each having distinct timescales of invasion. In all cases, $\mu _{\text {crit}}=12705$ according to (15). Alongside parameter values in Table 1, $\beta = 10^{11}$ and concentrations are rescaled by $c = 10^6$. Here, we do not neglect the important effects of primary nucleation, as they are instrumental in slowly decaying clearance. The $k_n=0$ approximation does not approximate the dynamics of (**a**) or (**b**) well when seeded, but it does approximate the system well if $\lambda (0)<\mu _{\text {crit}}$, shown by the dotted curves in (**d**)

If we remove clearance in this model, it becomes equivalent to the previous (constant clearance) system (11) leading to unbounded growth. Once clearance decays to the basal clearance capacity associated with maximal damage, the dynamics of the system align with those of (11). However, the system dynamics prior to the point at which $\lambda _i = \mu _i$ are markedly different, as we observe sigmoidal growth in toxic mass as local clearance is driven to the basal clearance capacity (Fig. 8).

### Coupling the microscopic model to transport

So far, we have developed a model suitable to describe the dynamics of oligomer concentrations within a single region. Next, we consider a system with multiple regions of interest connected through a network with diffusive transport between different regions. As described in Fornari et al. (2019) the network based brain modelling approach provides a computationally cheap approach whilst maintaining high accuracy in reproducing histopathological data, compared to continuum modelling approaches. The brain connectome is defined as a weighted graph ${\mathcal {G}}$ with *V* nodes (*V* for vertices) and *E* edges obtained from tractography of diffusion tensor images. Specifically, the brain network is constructed from 418 healthy human magnetic resonance images obtained through the Human Connectome Project (McNab et al. 2013), using the Budapest Reference Connectome v3.0 (Szalkai et al. 2017). From the tractography, we extract the weighted adjacency matrix $\textbf{A}$ and define the graph Laplacian $\textbf{L}$

28 $$\begin{aligned} L_{ij} = - A_{ij}+\sum _{j=1}^{V} A_{ij},\qquad i,j=1,\ldots ,V. \end{aligned}$$

There are other possible definitions of the graph Laplacian obtained by normalizing rows, columns, or both. However, this is the only graph Laplacian that has the property of preserving mass during transport and ensures that no transport occurs between two regions with the same concentration (for a detailed description and discussion see Brennan et al. 2025).

Incorporating the assumptions specified in the previous section, $k_n=0$ to approximate the seeded system, the truncated master equations for aggregates of size *i* at node $j=1,2,\cdots ,V$, including axonal transport, form an infinite system of first order ODES:

29a $$\begin{aligned}&{\frac{{\text {d}}{{m_j}}}{{\text {d}}t}} =- \rho _{1}\sum _{k=1}^V L_{jk} {m_{k}}, \end{aligned}$$

29b $$\begin{aligned}&{\frac{{\text {d}}{{p_{2,j}}}}{{\text {d}}t}} =- \rho _{2}\sum _{k=1}^V L_{jk} {p_{2,k}}-\lambda _{2,j} p_{2,j} + k_n m_j^2 -2 k_{+}m_j p_{2,j}+k_{2} \sigma (M_j) m_j^2 M_j,\end{aligned}$$

29c $$\begin{aligned}&{\frac{{\text {d}}{{p_{i,j}}}}{{\text {d}}t}} =- \rho _{i}\sum _{k=1}^VL_{jk} {p_{i,k}} -\lambda _{i,j} p_{i,j}+ 2 k_{+}m_j (p_{i-1,j}-p_{i,j}), \end{aligned}$$

29d $$\begin{aligned}&{\frac{{\text {d}}{{\lambda _{i,j}}}}{{\text {d}}t}} = f(\lambda _{i,j}), \end{aligned}$$

with initial conditions

30 $$\begin{aligned} m_i(0) = m_{i,0}, \quad p_{i,j}(0)=p_{i,j,0}. \end{aligned}$$

Here $\rho _i$ is the diffusion coefficient of the *i*-mer taken to be small or a function of *i* (Fornari et al. 2020; Bertsch et al. 2017). As before, the first two moments of the population distribution are

31 $$\begin{aligned} P_j=\sum _{i=2}^{\infty } p_{i,j},\quad M_j=\sum _{i=2}^{\infty } i p_{i,j}, \end{aligned}$$

representing the total number and total mass concentrations of aggregates in the region of interest (ROI) *j*, respectively. Taking clearance to be constant in time, the moment equations are given by:

32 $$\begin{aligned} &  {\frac{{\text {d}}{{M_j}}}{{\text {d}}t}} =-\sum _{i=2}^\infty \lambda _{i,j} i p_{i,j}- \rho _{j} \sum _{k=1}^VL_{jk} {M_{k}} +2 k_{n} m_j^2 +2 k_{+}m_j P_j+2 k_{2} \sigma (M_j) m_j^2 M_j, \nonumber \\ \end{aligned}$$

33 $$\begin{aligned} &  {\frac{{\text {d}}{{P_j}}}{{\text {d}}t}} = -\sum _{i=2}^\infty \lambda _{i,j} p_{i,j}- \rho _{j} \sum _{k=1}^VL_{jk} {P_{k}} + k_{n} m_j^2 + k_{2} \sigma (M_j) m_j^2 M_j. \end{aligned}$$

**Fig. 9:**
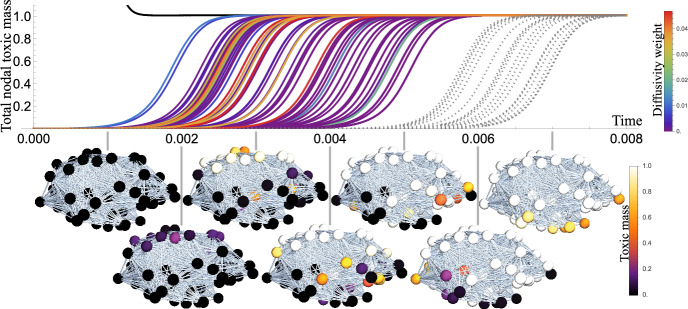
The order of A$\beta$ invasion of nodes on the connectome and the corresponding sagittal views of the spread on the brain network. Subcritical initial clearance of $\lambda (0)=10$ is uniform across nodes, and we impose an initial seed of $M(0) = m_0$ in the posterior cingulate, where A$\beta$ is first observed. Here in (29), $\rho =0.01$, $k_n=0$, and all other parameters are shown in Table 1. The top row shows the cascade of toxic proteins across nodes, with total toxic mass at each node normalized by the saturated concentration. For immediate neighbors of the seed nodes, diffusivity weightings are represented by color. Nodes at a path length of two from the seed nodes are shown by grey dashed curves, while biomarkers corresponding to the seed nodes are shown in black (color figure online)

The order of node invasion across the brain network suggests a direct applicability of the diagonalization results of Fornari et al. (2020) to our model. In Fig. 9, we observe immediate neighbors of the single seed node are invaded first, influenced heavily by the diffusivity weighting along the edges emanating from this seed. Subsequently, nodes of path length two are invaded, encompassing all nodes in the small-world network. Nodes with the lowest connectivity, such as the frontal pole, are invaded last. Figure 9 also displays the order of ROI invasion across the connectome and the corresponding network representation.

**Fig. 10:**
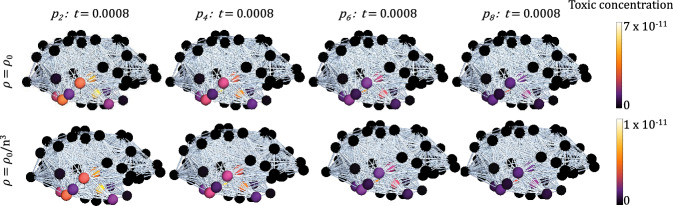
Sagittal views of the distribution of aggregates of increasing size on the brain network at the fixed time point $t=0.0008$. Here we seed in the entorhinal cortex $p_2 = m_0$, with constant clearance $\lambda = 10^3$ in (29). The difference between the top row and bottom row is constant diffusion $\rho =0.01$, and size-dependent diffusion $\rho = \rho _0/n^3$, where *n* is the oligomer size, resulting in lower toxic concentrations at $t=0.0008$ (see colour bar limits), but no change in the relative occupancy of aggregates

The growth of toxic mass across the connectome in this order is facilitated primarily by the transport of nuclei. To demonstrate this, first the distribution of oligomers of size $i=$ 2, 4, 6 and 8, at a fixed time point, is seen in Fig. 10 (first row) given a size-independent diffusion coefficient in (29). Aggregates spreading in travelling waves, and total toxic mass is a propagating front as described in Putra et al. (2023). It is observed experimentally (Nicholson et al. 2000; Goodhill 1997; Nicholson and Syková 1998) that in vivo aggregate transport scales with size. Specifically, large fibril assemblies do not move, and in previous models (Bertsch et al. 2017; Achdou et al. 2013) the diffusion coefficient of a soluble peptide is taken to scale as a reciprocal of the cube of its molecular weight.

Perhaps counter-intuitively, the distinction in progression through nodes being characterised by first low weight oligomers, and later by increasing aggregate sizes, is not exacerbated by the assumption of transport scaling with size, as seen by comparing rows in Fig. 10. The order of invasion and relative times of invasion remain the same, but the time taken to reach higher concentrations increases. This emphasises the role of nuclei spreading through the connectome, since local dynamics dominate over the delivery of aggregates by diffusion. We conclude that regardless of the form of size-dependent diffusivity, provided diffusion is small, the transport of larger aggregates does not significantly change the brain-wide dynamics.

## Applications to therapeutic modelling

Next, we investigate the impact of monoclonal antibodies (mAbs), a form of immunotherapy known to influence microscopic parameters in chemical kinetic models, on the aggregation and propagation of misfolded toxic proteins. Using the local and brain scale models derived and studied in the previous section, we analyse, replicate, and propose treatment strategies on the structural connectome based on results in vitro. Parameters extracted in vitro are scaled up to display the impacts on whole-brain neurodegeneration to simulate treatments in a fully mechanistic model at the whole-brain scale. Notably, FDA-approved drugs such as lecanemab, and drugs with promising phase III trial results, such as donanemab, operate by ultimately increasing the effectiveness of brain clearance. Thus, a model that captures the intricacies of the brain’s endogenous clearance and the enhancement of this mechanism from drugs is crucial.

**Fig. 11:**
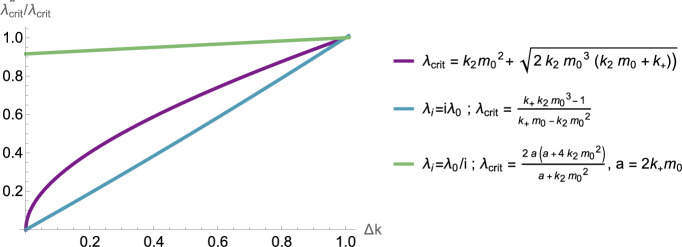
For varying $\tilde{k_2} = \Delta k k_2$, the ratio of critical clearance values in the presence of drugs $\tilde{\lambda }_{\text {crit}}$ and critical clearance associated with $\Delta k = 1$ (without drugs). Results are displayed for critical clearance formulae associated with different models, namely (11) with constant clearance $\lambda _i = \lambda$ (purple), and size-dependent clearance $\lambda _i = i \lambda _0$ (blue), and $\lambda _i = \lambda _0/i$ (green) (color figure online)

Linse et al. (2020) found, by fixed point solution method of the coarse-grained protein kinetic equations, the kinetic fingerprints of various mAbs. For example, using the method described in Meisl et al. (2016), they identified that aducanumab causes an apparent reduction of the free oligomer concentration by inhibiting the critical molecular process, secondary nucleation, through which oligomers form. With the highest dose of aducanumab (100 nM), corresponding to a substoichiometric molar ratio of 0.03:1 antibody: A$\beta _{42}$, there is a 69% reduction in the secondary nucleation rate constant. With the lowest dose tested (250 pM), there is still a 39% reduction. Thus one effect of the drug on the aggregation pathway can be expressed as a rate constant change in the presence of aducanumab:

34 $$\begin{aligned} \tilde{k_2} = \Delta k k_2, \end{aligned}$$

where $\Delta k \in [0,1]$ is dependent on the dose of the drug. Importantly, variations in $k_2$ reduce the critical clearance for all models, as seen in Fig. 11, with implications for disease timescales and propagation patterns as discussed in Brennan et al. (2023).

The inhibitory effect on secondary nucleation originates from the interaction of aducanumab with amyloid fibrils. Linse et al. (2020) observed a low affinity for monomers and a very high affinity (1nM) for fibrils, in agreement with previous findings (Arndt et al. 2018). Fibrils become fully coated with aducanumab along their entire length, effectively interfering with secondary nucleation at the fibril surface. Further, mAbs binding to amyloid fibrils targets them for microglia-mediated removal and enhances the clearance of plaques (Sevigny et al. 2016; Söderberg et al. 2023).

Mazer et al. (2023) model the effect of drugs solely through increased clearance in a compartmental model; the Q-ATN model. The pharmacodynamic (PD) drug effect of antibody-mediated plaque removal is quantified by a linear relationship between antibody concentration and clearance. The PD model assumes a pseudo-first-order rate constant for plaque removal ($\lambda _{\text {drug}}$) that is proportional to the plasma concentration ($C_p$), with an antibody-specific proportionality constant (*L*) given by

35 $$\begin{aligned} \lambda _{\text {drug}}(C_p) = L C_p. \end{aligned}$$

The *L* values were estimated by fitting the time course of mean amyloid PET data during treatment using a non-linear least squares algorithm.

We directly substitute drug inhibited parameters into the system (11) to obtain, in the case of aducanumab,

36a $$\begin{aligned}&\frac{{\text {d}}{{m}}}{{\text {d}}t} = 0, \end{aligned}$$

36b $$\begin{aligned}&\frac{{\text {d}}{{p_2}}}{{\text {d}}t} = -(\lambda _2+\lambda _{\text {drug}}) p_2 + k_{n} m^2 - 2 k_{+} m p_{2} + \tilde{k}_{2} \sigma (M) m^2 M, \end{aligned}$$

36c $$\begin{aligned}&\frac{{\text {d}}{{p_i}}}{{\text {d}}t} = -(\lambda _i+\lambda _{\text {drug}}) p_i + 2 k_{+} m (p_{i-1} - p_{i}), \quad i > 2 \end{aligned}$$

where, as before,

37 $$\begin{aligned} \sigma (M) = \frac{K_M }{K_M + M^{2}}, \quad P=\sum _{i=2}^{\infty } p_{i}, \quad M=\sum _{i=2}^{\infty } i p_{i}. \end{aligned}$$

**Fig. 12:**
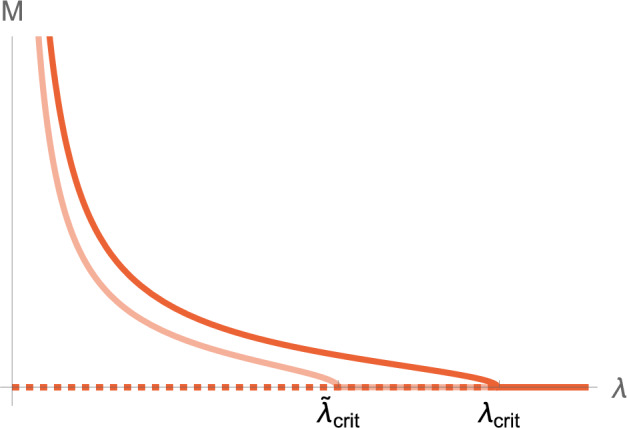
Unstable (dashed) and stable (solid) equilibrium solutions for total toxic mass $M_\infty$ are depicted without drugs (red) and in the presence of aducanumab (lighter red). The presence of aducanumab initiates a reduction in secondary nucleation to $\tilde{k_2} = 0.5 k_2$, causing the critical clearance reduces from $\lambda _{\text {crit}} = 12750$ to $\tilde{\lambda }_{\text {crit}} = 8750$ given by (15), and a reduction in the saturated toxic state $M_2$ given by (14) (color figure online)

Next, we consider the effects of aducanumab on only the kinetic parameters expressed through the reduced secondary nucleation rate constant $\tilde{k_2}$, identified to be the drug’s inhibitory action on the aggregation chain. This results in a new critical clearance, which varies with $k_2$ according to (15) (Fig. 11), and the steepness of the $M_2$ fixed state according to (14). The toxic mass equilibrium is shown in Fig. 12 in the presence of the kinetic action of aducanumab (orange) and without treatment (red). In addition to this kinetic effect, Mazer et al. (2023) suggest that the preferential binding of aducanumab to fibrils facilitates clearance at a rate proportional to drug concentration. Therefore, we model this effect by increasing aggregate size-specific clearance by $\lambda _{\text {drug}}$.

Coupling (36) with transport across the connectome using the graph Laplacian, as done in (29), the homogeneous system serves as a good approximation of the brain-scale dynamics. The progression through the brain network, with and without treatment, highlights the ability of monoclonal antibodies to localize the disease entirely to the initial seed nodes if caught within the clearance window of $\tilde{\lambda }{\text {crit}}< \lambda < \lambda {\text {crit}}$. Specifically, this is achieved by modeling the administration of aducanumab, which induces a reduction in secondary nucleation, $\tilde{k_2} = 0.5 k_2$, whereas in the untreated state, we observe brain-wide progression, as illustrated in Fig. 9. This dependence of drug efficacy on initial clearance, along with the characterization of the drug’s effects purely in terms of brain clearance, further underscores the pivotal role of clearance in neurodegeneration.

## Mathematically informed treatment strategies

Mathematical models provide a platform for experiments that would be otherwise difficult or impossible to conduct in humans. Here we study potential treatment strategies based on our network model of brain-scale aggregation with in vivo effects.

### Target clearance of smaller aggregates

Different drugs target aggregates of different sizes. We simulate this effect by assuming that clearance halts or reduces neurodegeneration. An enhanced clearance $\lambda _{\text {drug}}$, will specifically affect aggregates within a particular size interval, in addition to the natural (assumed to be initially subcritical) background clearance $\lambda _a$ of the human brain. To capture these effects in a local model representative of a single brain region in vivo like (11), we keep the clearance rates constant for most sizes but elevated dramatically across an interval $n_0 \le i \le n_1$:

38a $$\begin{aligned}&\frac{{\text {d}}{{m}}}{{\text {d}}t} = 0, \end{aligned}$$

38b $$\begin{aligned}&\frac{{\text {d}}{{p_2}}}{{\text {d}}t} = -\lambda _2 p_2 + k_{n} m^2 - 2 k_{+} m p_{2} + k_{2} \sigma (M) m^2 M, \end{aligned}$$

38c $$\begin{aligned}&\frac{{\text {d}}{{p_i}}}{{\text {d}}t} = -\lambda _i p_i + 2 k_{+} m (p_{i-1} - p_{i}), \quad i > 2,\end{aligned}$$

38d $$\begin{aligned}&\lambda _i ={\left\{ \begin{array}{ll} \lambda _a + \lambda _{\text {drug}}, & n_0 \le i \le n_1\\ \lambda _a, & \text {otherwise}, \end{array}\right. } \end{aligned}$$

where

39 $$\begin{aligned} \sigma (M) = \frac{K_M }{K_M + M^{2}}, \quad P=\sum _{i=2}^{\infty } p_{i}, \quad M=\sum _{i=2}^{\infty } i p_{i}, \end{aligned}$$

with the corresponding reduced moments system

40a $$\begin{aligned}&{\frac{{\text {d}}{{P}}}{{\text {d}}t}} \,\,= - \lambda _a P - \lambda _{\text {drug}}\sum _{i=n_1}^{n_2} p_{i} +k_{n}m_0^2+ k_{2}\, \sigma (M) m_0^2 M, \end{aligned}$$

40b $$\begin{aligned}&{\frac{{\text {d}}{{M}}}{{\text {d}}t}} = - \lambda _a M - \lambda _{\text {drug}}\sum _{i=n_1}^{n_2} i p_{i} + 2 k_{n}m_0^2+{2 k_{+}m_0 P} + {2}k_{2}\, \sigma (M) m_0^2 M. \end{aligned}$$

**Fig. 13:**
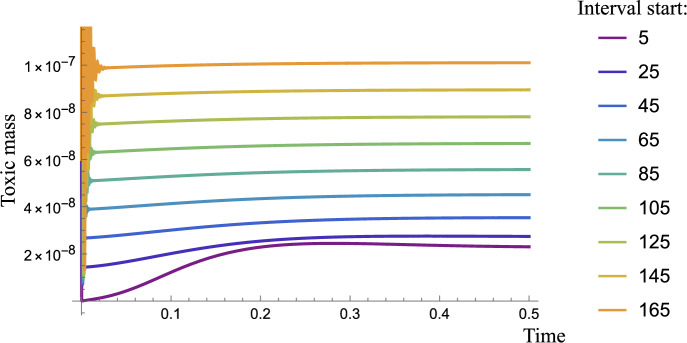
Total toxic mass evolution with heightened clearance as in (38d) with $n_0$ specified in the legend, and $n_1 = n_0+10$. The equilibrium solution for toxic mass $M_\infty$ is lower when smaller aggregates are targeted with heightened clearance. Here $\lambda _a = 10$ and $\lambda _{\text {drug}} = 10^5$ and parameters are as found in Table 1

As seen in Fig. 13, we perform a sweep of intervals $(n_0,n_1)$ and observe that targeting aggregates of smaller size decreases the total toxic mass in the system the most. This underscores the fundamental role of secondary nucleation in forming plaques and indicates that smaller aggregates constitute the largest toxic mass in the system.

An explanation of the observed results in Fig. 13 can be understood by considering the fixed point solutions. Specifically, a steady state $p_i^*$ for $i> 2$ satisfies the recurrence relation

41 $$\begin{aligned} p_i^* = \delta _i p_{i-1}^*,\quad \delta _i = \frac{2k_+ m_0}{\lambda _i + 2k_+ m_0},\quad i>2, \end{aligned}$$

where each recursive effect is dependent on *i*. Consequently, the fixed point of the *i*-mer is

42 $$\begin{aligned} p_i^* = \triangle _i p_2^*, \quad \text {where } \quad \triangle _i = {\left\{ \begin{array}{ll} \prod _{j=3}^i \delta _j & \text {for } i>2, \\ 1 & \text {for } i = 2. \end{array}\right. } \end{aligned}$$

Removing *i*-mers of sizes $n_1\le i \le n_2$ gives the toxic mass fixed point,

43 $$\begin{aligned} M_* = \triangle p_2^* - \sum _{k=n_1}^{n_2} k \triangle _k p_2^*, \end{aligned}$$

where we define $\triangle = \sum _{k=2}^\infty k \triangle _k$, and

44 $$\begin{aligned} p_2^*= \frac{\sqrt{23} \sqrt{-\lambda _2 - 2 k_{+} m_0 + k_2 m_0^2 \triangle }}{1000 \sqrt{ \lambda _2 \triangle ^2 + 2 k_p m_0 \triangle ^2}}. \end{aligned}$$

It remains to prove that

45 $$\begin{aligned} \sum _{k=n_1}^{n_2} k \triangle _k p_2^* > \sum _{k=n_3}^{n_4} k \triangle _k p_2^*, \end{aligned}$$

or

46 $$\begin{aligned} (a+\lambda n_1)\Big ( \frac{a}{a + \lambda } \Big )^{n_1} - (a+\lambda n_2)\Big ( \frac{a}{a + \lambda } \Big )^{n_2} > (a+\lambda n_3)\Big ( \frac{a}{a + \lambda } \Big )^{n_3} - (a+\lambda n_4)\Big ( \frac{a}{a + \lambda } \Big )^{n_4}, \end{aligned}$$

for $n_1<n_2<n_3<n_4$ where $a = 2 k_+ m_0$. Expanding the brackets, to leading order

47 $$\begin{aligned} n_1 \xi ^{n_1} - n_2 \xi ^{n_2} > n_3 \xi ^{n_3} - n_4 \xi ^{n_4}, \end{aligned}$$

where $\xi = a/\lambda$. Since there is a fixed difference between intervals, say *d*,

48 $$\begin{aligned} n_1 \xi ^{n_1} - n_2 \xi ^{n_2} > \xi ^{d} ((n_1+d) \xi ^{n_1} - (n_2+d) \xi ^{n_2} ), \end{aligned}$$

so we require

49 $$\begin{aligned} (n_1 - \xi ^{d} (n_1+d))\xi ^{n_1}> (n_2 - \xi ^{d} (n_2+d))\xi ^{n_2}. \end{aligned}$$

Further, there is also a fixed interval size $n_2 = n_1 + n_d$ so

50 $$\begin{aligned} (n_1 - \xi ^{d} (n_1+d))\xi ^{n_1}> (n_1 - \xi ^{d} (n_1+d))\xi ^{n_1}\xi ^{n_d} + (n_d - \xi ^{d} (n_d+d))\xi ^{n_1}\xi ^{n_d}, \end{aligned}$$

that is,

51 $$\begin{aligned} \xi ^{n_d} \Bigg ( 1 + \frac{n_d - \xi ^{d} (n_d+d)}{n_1 - \xi ^{d} (n_1+d)} \Bigg ) < 1, \end{aligned}$$

which holds true since $\xi \ll 1$. Hence, in the constant clearance regime, removing intervals of smaller aggregates reduces the total toxic mass equilibrium more than removing the same-sized intervals of larger aggregates. It remains to explore the effect of removing larger intervals of larger aggregates and a critical interval size, which would be more valuable than removing lower-weight oligomers.

Given the pronounced toxicity of oligomeric intermediates relative to larger fibrillar species, actively eliminating oligomers has a dual impact. By targeting smaller aggregates, we significantly reduce the total toxic mass to the greatest extent possible. This approach aligns with our primary objective of addressing the most toxic species generated during aggregation. As a reference, aducanumab partially targets oligomers but mostly clears insoluble amyloid plaques (Tolar et al. 2020).

### Optimal drug administration strategies

A variety of dosing strategies can be analysed and simulated directed in the nucleation-aggregation-clearance model with in vivo effects (11). In this section, we formulate an optimisation problem with the objective of minimising the accumulation of toxic mass in a given time period, over the parameters of the functional form of drug-induced clearance $\lambda (t)$. This allows us to determine the optimal frequency and volume of doses, while adhering to drug toxicity constraints, in the context of a computational drug trial.

Assuming a linear relationship between antibody concentration and clearance as proposed by Mazer et al. (2023), we take the concentration of the drug administered to be directly proportional to the drug-enhanced clearance increment $\lambda _{\text {drug}}$. That is, $\lambda _{\text {drug}}(C_p) = L C_p$ where $C_p$ is drug concentration and *L* is constant. In the first instance, we assume that drug administration is spaced equally and decays exponentially, being cleared from the brain as a non-aggregating particle. Emulating the drug concentration profiles described in Mazer et al. 2023, [Figure 1], we simulate a dosing regime through the following clearance profile:

52 $$\begin{aligned} \lambda (t) = \lambda _{\text {drug}} e^{-A\mod (t,\,B)} + \lambda _a, \end{aligned}$$

in (11). Here *A* is the clearance rate of the drug from the brain, *B* is the time period (days) between drug doses, $\lambda _a$ is the background natural clearance, and $\lambda _{\text {drug}}$ is the drug-enhanced increase in clearance that is assumed to be proportional to the dose.

**Fig. 14:**
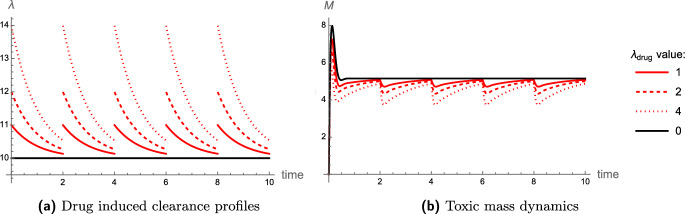
The drug induced clearance profiles (a) according to (52), with $\lambda _a = 10$, $B=2$ and various $\lambda _{\text {drug}}$ as specified in the legend. The no drug case is displayed by the black curves. The corresponding toxic mass *M* evolution shows a sinusoidal steady state in the presence of drugs. The system is (13) with parameter values as in Table 1

To demonstrate the effects of this dosing regime, clearance profiles (52) and the response in toxic mass evolution according to (11) are displayed in Fig. 14 corresponding to three dosing strategies. In this example, for illustrative purposes, the black curves show the no-treatment case, and only $\lambda _{\text {drug}}$ (the dosage) varies across strategies with fixed $A = 1$, $B=2$, and ambient clearance $\lambda _a = 10$. We observe a sinusoidal steady state in the presence of drugs, with the steady state’s peak reducing and the steady state’s range increasing, with $\lambda _{\text {drug}}$ increasing.

Considering the effects of varying both *B* and $\lambda _{\text {drug}}$ on misfolded protein mass, herein referred to as the *regime parameters*, the best strategy is to maximise the frequency and volume of treatments. However, there is a cost involved in raising $\lambda _{\text {drug}}$ (dosage) and reducing *B* (time between each dose) due to the toxicity of antibodies to the brain environment, such as a significant risk of amyloid-related imaging abnormality (ARIA) (Withington and Turner 2022; Cummings et al. 2021). With this in mind, the following optimisation problem naturally arises.

Given a trial period of say $t=28$ days, we aim to identify the optimal number of days between drug administration *B* and volume of drugs given that is proportional to drug-induced clearance $\lambda _{\text {drug}}$. We supplement the problem with the constraint that the total mass of drugs (mg) administered during this period invokes a limited integrated clearance increment, denoted $C_{\text {max}}$. For simplicity, we choose $A=1$, assuming that *A* remains constant as clearance increases. We optimise $\lambda _{\text {drug}}$ and *B* such that the average toxic mass across one cycle of drug administration, i.e. the average of the steady states shown in Fig. 14b, $\bar{M}$, is minimised. In full, the optimisation problem reads

53 $$\begin{aligned}&\text {Minimise} \quad \bar{M} \quad \text {over} \quad 0.2<B<t_{\text {max}}, \quad 0.2<\lambda _{\text {drug}}<80 \end{aligned}$$

54 $$\begin{aligned}&\text {subject to} \end{aligned}$$

55 $$\begin{aligned}&\int _0^{t_{\text {max}}} (\lambda _{\text {drug}} e^{-A\mod (t,\,B)}) \, \text {d}t = C_{\text {max}}. \end{aligned}$$

**Fig. 15:**
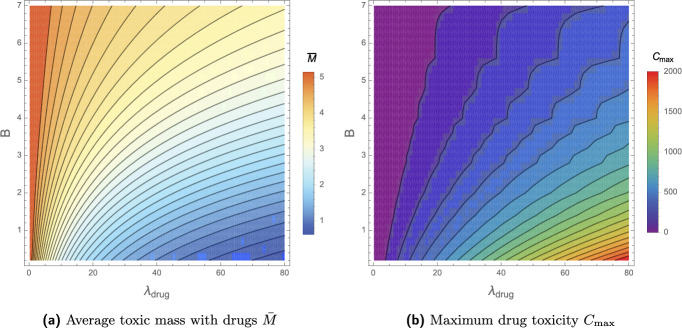
Contour maps showing the average toxic mass $\bar{M}$ (**a**) and toxic load of the drug $C_{\text {max}}$ (**b**) as functions of the drug dosing regime parameters in (52): the drug induced maximum clearance increment $\lambda _{\text {drug}}$, and the frequency of treatments *B*

**Fig. 16:**
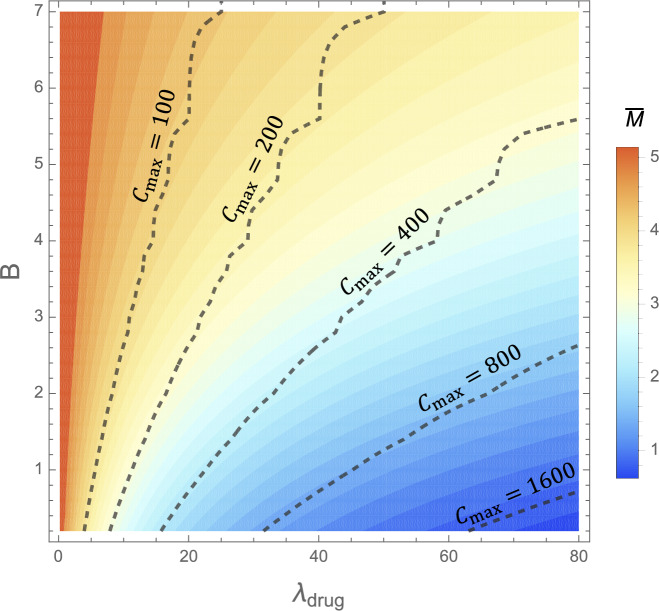
Contour plot with the colours displaying the average toxic mass $\bar{M}$ accumulated in the presence of a drug with different dosing regimes defined by varying parameters $\lambda _{\text {drug}}$ correlated directly with dosage size, and *B* the frequency of doses. Toxic mass is found numerically by solving (11) with (52). Solutions which satisfy the constraints outlined in (55), namely with regime parameters invoking the specific toxic load $C_{\text {max}}$, for different values of $C_{\text {max}}$, lie along the contour lines

Contour plots of the average toxic mass $\bar{M}$ for varying regime parameters are shown in Fig. 15a, with the corresponding drug toxicity $C_{\text {max}}$ shown in Fig. 15b. We identify the optimal dosing regime, subject to a specific drug toxicity constraint $C_{\text {max}}$ by consulting the combination of Fig. 15a and b in Fig. 16. Solutions satisfying the constraints $C_{\text {max}}\approx 100,\,200,400,800,1600$ in (55) lie along the respective dashed lines, and the colour function displays the relative average toxic mass equilibrium $\bar{M}$. Subject to these constraints, solutions with the lowest average toxic mass $\bar{M}$ correspond to the lowest spacing between doses, $B\rightarrow 0$.

The optimal strategy, therefore, is the trivial one: to take $B\rightarrow 0$, corresponding to a constant supply of drugs. Of course, this could be impractical or detrimental to a patient’s quality of life. Given the potential impracticality of constant drug supply, the key question is at what cost, in terms of toxic mass $\bar{M}$, can drug doses be distributed (increasing *B*). For reference, consider regime parameters corresponding to $C_{\text {max}} = 100$ in (55). The optimal solution, a constant supply of drugs with $\lambda _{\text {drug}}= 100/28$, results in the minimum allowable average toxic mass of $\bar{M}=3.79$. Other solutions satisfying the same drug toxicity constraint, but not necessarily minimising $\bar{M}$, are shown in Fig. 16 and demonstrate some flexibility in dosing strategies. For example, $\lambda _{\text {drug}} = 5.6$ once a day results in the only slightly increased $\bar{M}=3.81$. A solution with seven days between doses of $\lambda _{\text {drug}} = 25$ results in a similar average toxic steady state of $\bar{M}=4.22$, albeit with a higher variance around this equilibrium. This implies some leniency on dosing frequency. Overall, as seen in Fig. 16, $\bar{M}$ does not greatly vary along the constant $C_{\text {max}}$ contours; the contours of $\bar{M}$ and $C_{\text {max}}$ in Fig. 15a and b follow similar trends. The amount that $\bar{M}$ varies with *B* with $C_{\text {max}}$ constant is dependent on $C_{\text {max}}$, as observed by the higher range in $\bar{M}$ values along the $C_{\text {max}}=400$ contour compared to the $C_{\text {max}}=100$ contour. Therefore, depending on $C_{\text {max}}$ and the desired reduction in toxic mass, increasing the days between doses can lead to insignificant increases in toxic mass, with potentially significant improvements in quality of life.

**Fig. 17:**
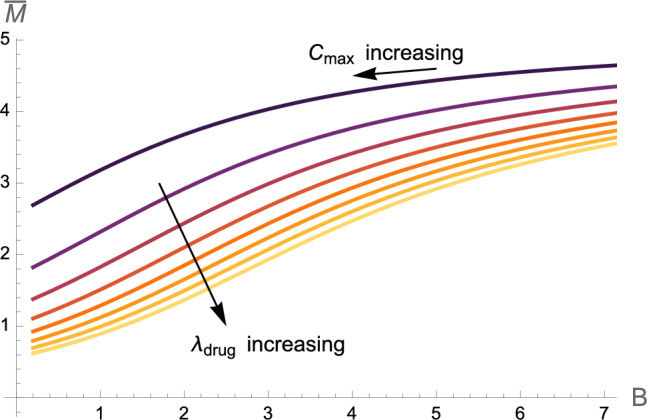
The evolution of the average toxic mass $\bar{M}$ with constant drug dosage corresponding to a clearance increment of $\lambda _{\text {drug}}$ and varying frequencies of administration *B*. Constant $\lambda _{\text {drug}}$ values range from 10 to 80 with differences of 10 between curves

In addition, as shown by Fig. 17, keeping $\lambda _{\text {max}}$ constant and lowering *B* can have a high impact on reducing $\bar{M}$ for smaller values of *B* but is less impactful for higher *B*. Further, increasing $\lambda _{\text {drug}}$ is less impactful for higher values of $\lambda _{\text {drug}}$ (shown by the lighter curves). Together, these results hold potential in determining dosing strategies in personalised medicine, with initial conditions of (11) reflecting the initial toxic loads of patients and parameters in (52) tunable to the desired reduction in toxic load.

## Discussion

Theoretical research into toxic mass accumulation in neurodegenerative diseases has thus far exclusively consisted of either detailed in vitro analysis of aggregation kinetics or in vivo studies on the effects of transport and clearance in macroscale models on networks, with the former being validated against experimental observations and the latter with structural data. Both aspects have proved essential for our understanding of AD pathology. However, there is a cloudy middle ground restricting our understanding of aggregation inside the complex domain of the human brain. The development of a nucleation-aggregation model which includes in vivo effects such as protein production, saturation, transport and, most importantly, the clearance of solutes, remains a task that has only been addressed mechanistically in two prior studies (Fornari et al. 2020; Thompson et al. 2021). Fornari et al. (2020) used a Smoluchowski-type model to analyse the effect of aggregate transport at the brain scale, while Thompson et al. (2021) considered the effects of a bulk constant clearance at the microscopic scale. Our work combines both approaches within the same mathematical framework, establishing a general picture for the self-assembly of A$\beta$ in the aggregation cascade in vivo across the connectome. We have derived a class of nucleation-aggregation-clearance models for the homogeneous dynamics and spatial progression of A$\beta$ in the brain, tracking the evolution of different size aggregates with distinct properties instrumental in neurodegeneration. This new class of models provides a therapeutic modelling platform reflective of the human brain for the simulation of potential therapies at the spatial and temporal scales of the disease. Notably, we reveal the therapeutic effect of enhanced clearance behind the success of recent drug trials. The dependence of drug efficacy on initial clearance, along with the characterisation of the drug’s effects purely in terms of clearance, further emphasises the pivotal role of clearance in neurodegeneration.

### Brain scale models of nucleation, aggregation, clearance and transport

The first primary aim of this work was to develop a comprehensive and entirely mechanistic model encompassing the aforementioned in vivo effects, along with physiological effects not previously modeled, with a focus on quantifying the role of dynamic clearance of aggregates in neurodegeneration at the brain scale. To address the complexities of developing a model representative of a single brain region, we advance current state-of-the-art chemical kinetic models by incorporating physiological effects observed in experiments. Then, we include strong transport anisotropy in the brain through the graph Laplacian to simulate aggregate dynamics across the spectrum of aggregate sizes at the brain scale. The study of such systems is guided by the homogeneous case for which both total mass evolution and aggregates’ size distribution are obtained analytically, and bifurcation analysis reveals critical clearance regimes dependent on kinetic rate constants.

At the local ROI level, analysis of the homogeneous nucleation-aggregation-clearance models (11) reveals that the clearance rate dictates the timescale of the nucleated transition to an aggregated brain region and the final toxic mass equilibrium. In the absence of a simulated seed, an increase in toxic mass is characterised by an early phase of seeding depending on primary nucleation, followed by a period of linear growth mostly controlled by aggregation of monomers onto the fibril, and then a phase of autocatalytic growth dominated by the multiplicative processes of secondary nucleation and elongation. Once enough toxic mass accumulates, clearance dominates, and the toxic mass approaches a saturated steady state. Importantly the critical clearance rate, above which all aggregates clear from a brain region, holds significant therapeutic potential. Recovering similar results to Thompson et al. (2021), the critical clearance formulae depend upon the assumed clearance form relative to aggregate size, with different dependencies on the aggregation rate constants. When analysing the local system with dynamic clearance given by (26), local clearance reduces to the basal clearance capacity at rates relative to the difference between initial clearance and basal clearance. After this point the dynamics of the system are equivalent to those of the homogeneous nucleation-aggregation-clearance models (11) so we recover the same critical *basal* clearance regimes as for clearance in (11).

To model the interplay between aggregation and clearance at the brain-scale, the local dynamics are coupled with anisotropic transport across the connectome in (29). As identified first by Fornari et al. (2020), the progression of the disease consists of an initial stage that develops at the seeded node, followed by primary infection in connected nodes, mainly driven by diffusion rather than aggregation kinetics. These nodes distribute seeds, and due to the brain’s small-world network structure, secondary infection develops in most nodes soon after primary infection. In the growth-dominated regime representative of the toxic protein cascade in a human brain, local dynamics dominate once a node receives a seed by primary or secondary infection. We thus find that the analytical results of the homogeneous model (11) are a good approximation for the connected dynamics at the brain scale. Furthermore, the brain-scale dynamics appear invariant to the diffusion of aggregates larger than nuclei, indicating that the transport of dimers is the primary factor driving pathology, and size-dependent diffusion does not significantly alter the dynamics.

### Therapeutic modelling insights

The pivotal field of Alzheimer’s drug design commands extensive research activity. Yet, drug trials and drug performance projections rely on data without providing comprehensive insights into the mechanisms of drug action. Current microscopic chemical kinetic models shed light on how drugs influence the aggregation process (Linse et al. 2020), while empirical compartmental models project the longer-term effects of anti-amyloid treatments by fitting data from clinical trials to semi-mechanistic macroscale models (Mazer et al. 2023), with no consideration of the drug action at the microscale. Our in vivo nucleation-aggregation-clearance model aims to bridge the gap between these two approaches, extending in vitro observations to decades of AD pathology at the brain scale, considering both the increase in clearance due to drugs and the inhibition of specific steps in the aggregation process. We thus present the first fully mechanistic model of brain-scale in vivo aggregation growth, clearance and transport that can describe the effects of therapeutic agents both analytically and through direct simulation, aid drug performance projection, and lead exploratory theoretical studies to ultimately guide drug design. We thus quantified the action of potential drugs on protein aggregation in vivo, and conducted a preliminary study on the most favorable treatment strategies, concerning optimal aggregate sizes to target, specific microscopic steps of aggregation to inhibit, and the frequency and volume of antibody dosage.

Specifically, we modeled the mechanism of action of treatments in the homogeneous system (36) and its corresponding brain-scale extensions to see the effect of the antibodies on the disease dynamics. For aducanumab, we modeled the pharmacodynamic property of a decreased secondary nucleation rate constant due to the drug binding to fibrils, thus inhibiting the aggregation process (Linse et al. 2020) and mediating enhanced clearance, as considered by Mazer et al. (2023). First focusing solely on the kinetic drug effects we observe a consistent pattern across all models: a decrease in critical clearance, leading to an improvement in the effectiveness of compromised local clearance. This enables a patient under treatment to completely transition to a healthy state or experience a lessened toxic load, depending on the initial clearance at the time of commencing therapy. Mathematically, the inhibitory actions of potential therapeutic agents on the aggregation process transforms the aggregated equilibrium (Fig. 12). Thus through steady-state analysis, we identify that the primary therapeutic effect of the drugs is a reduction in critical clearance, increasing the system’s overall *effective clearance*.

Considering the drug’s effect in the microscopic aggregation model (36) coupled to transport allows us to track the response in the evolution of distinct aggregate sizes across the connectome. The effects of the kinetic action of treatment are the same: a transformation of the toxic steady-state solution in the presence of drugs (Fig. 12), with the potential to initiate a transition from an unhealthy patient state ($\lambda <\lambda _{\text {crit}}$) to a healthy state ($\lambda >\tilde{\lambda }_{\text {crit}}$) in which the otherwise compromised clearance rates are sufficient to completely remove toxic mass in the presence of drugs. The complete inhibition of aggregation and propagation shown in ?? is due only to the kinetic action of the drug reducing $\lambda _{\text {crit}}$ increasing the effectiveness of the brains damaged clearance mechanisms. This transition from an unhealthy state to a healthy equilibrium is dependent upon the brain’s initial clearance upon drug administration, underscoring the need for treatments early on in disease progression when $\tilde{\lambda }_{\text {crit}}< \lambda <\lambda _{\text {crit}}$. The impact of drugs such as aducanumab on secondary nucleation thus has a profound effect, employing a decrease in the aggregation kinetic rate constants thereby improving the effectiveness of brain clearance. Our most critical finding is that the drug’s kinetic effect alone can be sufficient for a transition from a brain with detrimental levels of toxic mass to a healthy state. In addition, considering enhanced clearance due to drug binding in combination with the kinetic effect of the drugs leads to a more pronounced reduction in long-term toxic mass accumulation. Significantly, in the presence of drugs, the critical toxic seed as discussed in Brennan et al. (2023) is increased, so it is less likely that a dynamic clearance will drop below critical. For A$\beta$, such results at the local level have significant implications at the brain scale because, by the time the disease becomes symptomatic, A$\beta$ has likely spread throughout the brain and levels are approximately homogeneous. In future work, when modeling tau, it is integral to consider how treatment affects tau transport, as this is essential for reproducing the Braak stages.

### Limitations and extensions

In adapting the well-established chemical kinetic model to an in vivo setting, we have made several necessary modeling assumptions. While future scientifc advances may allow for refinement of these assumptions, they are currently essential for progress in understanding aggregation processes in the brain:

- The model presently overlooks the spatial organization of aggregates at the microscale, distinguishing oligomers and protofibrils purely based on size, rather than molecular structure or functional characteristics. To take these effects into account would require a better understanding of the shape of different oligomers.
- We assume a nucleus size of two monomers. While this assumption can be easily modified, it is not clear what the exact seeding size is or if there is a single one rather than a distribution. Larger seeding size would lead to an extended lag phase in the toxic mass dynamics predicted by our model.
- A key assumption in our model is that secondary nucleation saturates with the toxic mass *M*, which is crucial for achieving toxic mass saturation. This assumption might later be refined to account for saturation with brain volume. We also assume homeostasis in monomer production and clearance, leading to a constant monomer concentration, as suggested by clinical literature. It would be valuable to investigate variations in monomer production in future work, particularly to model treatments that inhibit amyloid precursor protein (APP).
- One of the primary challenges in this study is that the kinetic parameters are derived from in vitro data. Determining these rate constants within the human brain environment would be a significant step forward. Furthermore, clearance plays a critical role in slowing disease progression, and a detailed analysis of the clearance dynamics, particularly in relation to factors such as the vasculature and the glymphatic system, will be essential for uncovering fundamental mechanisms and identifying potential therapeutic interventions.

### Conclusion

Despite its limitation, our brain-scale nucleation-aggregation-clearance models open up avenues for the mathematically-informed therapeutic strategies to control pathological protein aggregation, addressing questions that current modelling studies fail to address at the brain scale. We have identified that a reduction in $\lambda _{\text {crit}}$ is successful in curing patients. Quantifying the actions of drugs in this way allows us to identify which rate constants might inhibit aggregation to provide the largest reduction in $\lambda _{\text {crit}}$. For example, if considering the size dependant clearance $\lambda _i = \lambda _0/i$ to be reflective of the human body, the form of the critical clearance rate (25) suggests that targeting the elongation processes would be most effective in reducing the critical clearance and most vitally translating the fixed point solution curve. Further, we performed a sweep of an interval of increased clearance and uncovered that targeting smaller aggregates is more impactful than targeting larger ones. Additionally, we studied an optimization problem to demonstrate how the models can inform dosing regimens, as described and simulated in Mazer et al. (2023). The results suggest that more frequent dosing is preferable for reducing average toxic mass and variance, although combinations of higher, less frequent doses can achieve the same steady states. A more in-depth analysis of the application of these models for pharmacological use is of great interest for future studies.

## Data Availability

The manuscript has no associated data.
